# Species conservation profiles of a random sample of world spiders III: Oecobiidae to Salticidae

**DOI:** 10.3897/BDJ.6.e27004

**Published:** 2018-08-02

**Authors:** Sini Seppälä, Sérgio Henriques, Michael L Draney, Stefan Foord, Alastair T Gibbons, Luz A Gomez, Sarah Kariko, Jagoba Malumbres-Olarte, Marc Milne, Cor J Vink, Pedro Cardoso

**Affiliations:** 1 Finnish Museum of Natural History, University of Helsinki, Helsinki, Finland Finnish Museum of Natural History, University of Helsinki Helsinki Finland; 2 IUCN SSC Spider & Scorpion Specialist Group, Helsinki, Finland IUCN SSC Spider & Scorpion Specialist Group Helsinki Finland; 3 University College London, London, United Kingdom University College London London United Kingdom; 4 University of Wisconsin-Green Bay, Green Bay, United States of America University of Wisconsin-Green Bay Green Bay United States of America; 5 University of Venda, Venda, South Africa University of Venda Venda South Africa; 6 University of Nottingham, Nottingham, United Kingdom University of Nottingham Nottingham United Kingdom; 7 Universidad Nacional de Colombia, Bogotá, Colombia Universidad Nacional de Colombia Bogotá Colombia; 8 Museum of Comparative Zoology, Harvard University, Cambridge, United States of America Museum of Comparative Zoology, Harvard University Cambridge United States of America; 9 University of Copenhagen, Copenhagen, Denmark University of Copenhagen Copenhagen Denmark; 10 University of Barcelona, Barcelona, Spain University of Barcelona Barcelona Spain; 11 University of Indianapolis, Indianapolis, United States of America University of Indianapolis Indianapolis United States of America; 12 Canterbury Museum, Christchurch, New Zealand Canterbury Museum Christchurch New Zealand

**Keywords:** Araneae, Arthropoda, conservation, endangered species, extinction risk, geographical range, IUCN.

## Abstract

**Background:**

The IUCN Red List of Threatened Species is the most widely used information source on the extinction risk of species. One of the uses of the Red List is to evaluate and monitor the state of biodiversity and a possible approach for this purpose is the Red List Index (RLI). For many taxa, mainly hyperdiverse groups, it is not possible within available resources to assess all known species. In such cases, a random sample of species might be selected for assessment and the results derived from it extrapolated for the entire group - the Sampled Red List Index (SRLI). The current contribution is the third in four papers that will constitute the baseline of a future spider SRLI encompassing 200 species distributed across the world.

**New information:**

A sample of 200 species of spiders were randomly selected from the World Spider Catalogue, an updated global database containing all recognized species names for the group. The 200 selected species where divided taxonomically at the family level, and the familes were ordered alphabetically. In this publication, we present the conservation profiles of 58 species belonging to the famillies alphabetically arranged between Oecobiidae and Salticidae, which encompassed Oecobiidae, Oonopidae, Orsolobidae, Oxyopidae, Palpimanidae, Philodromidae, Pholcidae, Pisauridae, Prodidomidae and Salticidae.

## Introduction

The IUCN Red List of Threatened Species is the most widely used information source on the extinction risk of species (Lamoreux et al. 2003, Rodrigues et al. 2006, Mace et al. 2008 but see Cardoso et al. 2011, Cardoso et al. 2012). It is based on a number of objective criteria, which are relatively easy to apply when adequate information is available (IUCN 2001). The Red List has been used to raise awareness about threatened species, guide conservation efforts and funding, set priorities for protection, measure site irreplaceability and vulnerability and influence environmental policies and legislation (Gardenfors et al. 2001, Rodrigues et al. 2006, Mace et al. 2008, Martín-López et al. 2009).

One of the uses of the Red List is to evaluate and monitor the state of biodiversity and a possible approach for this purpose is the Red List Index (RLI). The RLI helps to develop a better understanding of which taxa, regions or ecosystems are declining or improving their conservation status. It provides policy makers, stakeholders, conservation practitioners and the general public with sound knowledge of biodiversity status and change, and tools with which to make informed decisions. The RLI uses weight scores based on the Red List status of each of the assessed species. These scores range from 0 (Least Concern) to 5 (Extinct/Extinct in the Wild). Summing these scores across all species, relating them to the worst-case scenario - all species extinct, and comparing two or more points in time gives us an indication of how biodiversity is doing. At a global level, the RLI has been calculated for birds (Butchart et al. 2004, Hoffmann et al. 2010), mammals (Hoffmann et al. 2011), amphibians (Hoffmann et al. 2010), corals (Butchart et al. 2010), and cycads (United Nations 2015).

For many taxa, mainly hyperdiverse groups, it is not possible within available resources to assess all known species. In such cases, a random sample of species might be selected for assessment and the results derived from it extrapolated for the entire group - the Sampled Red List Index (SRLI, Baillie et al. 2008). The SRLI is now being developed for plants (Brummitt et al. 2015) and efforts towards a SRLI of butterflies (Lewis and Senior 2010) and Odonata are also in progress (Clausnitzer et al. 2009).

Spiders currently comprise over 47000 species described at a global level (World Spider Catalog 2018). Of these, only 199 species (0.4%) have beed assessed (www.redlist.org), of which the vast majority are from the Seychelles Islands or belong to the golden-orb weavers, Nephilidae (e.g. Kuntner et al. 2017). To these, a large number will be added in the near future, such as 55 species endemic to the Madeira and Selvagens archipelagos and 25 endemic to the Azores, all in Portugal (Cardoso et al. 2017, Borges et al. submitted). The vast majority of spiders assessed to date are therefore either regionally or taxonomically clustered and do not represent the group as a whole. The current contribution is the second in four papers (Seppälä et al. 2018a, Seppälä et al. 2018b) that will constitute the baseline of a future spider SRLI encompassing 200 species distributed across the world. All the assessments will in the future be included in the IUCN Red List of Threatened Species (www.redlist.org).

## Methods

A sample of 200 species of spiders were randomly selected from the World Spider Catalog (2018), an updated global database containing all recognized species names for the group. The 200 selected species where divided taxonomically to the family level, and those familes were ordered alphabetically. In this publication we present the conservation profiles of 58 species belonging to the famillies alphabetically arranged between Oecobiidae and Salticidae, which encompassed Oecobiidae, Oonopidae, Orsolobidae, Oxyopidae, Palpimanidae, Philodromidae, Pholcidae, Pisauridae, Prodidomidae and Salticidae.

Species data were collected from all taxonomic bibliography available at the World Spider Catalog (2018), complemented by data in other publications found through Google Scholar and georeferrenced points made available through the Global Biodiversity Information Facility (www.gbif.org) and also other sources (https://www.biodiversitylibrary.org; https://login.webofknowledge.com; http://srs.britishspiders.org.uk; http://symbiota4.acis.ufl.edu/scan/portal; https://lepus.unine.ch; http://www.tuite.nl/iwg/Araneae/SpiBenelux/?species; https://atlas.arages.de; https://arachnology.cz/rad/araneae-1.html; http://www.ennor.org/iberia). Whenever possible, with each species record we also collected additional information, namely habitat type and spatial error of coordinates.

For all analyses we used the R package red - IUCN redlisting tools (Cardoso 2017). This package performs a number of spatial analyses based on either observed occurrences or estimated ranges. Functions include calculating Extent of Occurrence (EOO), Area of Occupancy (AOO), mapping species ranges, species distribution modelling using climate and land cover, calculating the Red List Index for groups of species, among others. In this work, the EOO and AOO were calculated in one of two ways:

- for extremely range restricted species for which we assumed to know the full range, these values were classified as observed, the minimum convex polygon encompassing all observations used to calculate the EOO and the 2 km x 2 km cells known to be occupied used to calculate the AOO. When the EOO was smaller than the AOO, it was made equal as per the IUCN guidelines (IUCN Standards and Petitions Subcommittee 2017).

- for widespread species or those for which we did not have confidence to know the full range, we performed species distribution modelling (SDM). This was done based on both climatic (Fick and Hijmans 2017) and landcover (Tuanmu and Jetz 2014) datasets, at an approximately 1x1 km resolution. Before modelling, the world layers were cropped to the region of interest to each species and reduced to four layers through a PCA to avoid overfitting. In addition, latitude and longitude were used as two extra layers to avoid the models to predict presences much beyond the known region following the precautionary principle. We then used the Maxent method (Phillips et al. 2006) implemented in the R package red. Isolated patches outside the original distribution polygon were excluded from maps to avoid overestimation of EOO and AOO values. All final maps and values were checked and validated by our own expert opinion. KMLs derived from these maps were also produced using the red package. The cells (2x2 km) predicted to be occupied were used to calculate the AOO. When the EOO was smaller than the AOO, it was made equal as per the IUCN guidelines (IUCN Standards and Petitions Subcommittee 2017).

To infer on possible changes in range and/or abundance, and for forest species only, we have also consulted the Global Forest Watch portal (World Resources Institute 2014), looking for changes in forest cover during the last 10 years that could have affected the species.

Species sizes are total body size in mm and include the range for both males and females when known.

## Supplementary Material

Supplementary material 1 Distribution of *Oecobiusrivula* Shear, 1970 Data type: Distribution
File: oo_171413.kml Cardoso, P.

Supplementary material 2 Distribution of *Oecobiustrimaculatus* O. Pickard-Cambridge, 1872 Data type: Distribution
File: oo_203757.kml Cardoso, P.

Supplementary material 3 Distribution of *Caecoonopscubitermitis* Benoit, 1964 Data type: Distribution
File: oo_171165.kml Cardoso, P.

Supplementary material 4 Distribution of *Oonopinusaurantiacus* Simon, 1893 Data type: Distribution
File: oo_171194.kml Cardoso, P.

Supplementary material 5 Distribution of *Oonopstectulus* Chickering, 1970 Data type: Distribution
File: oo_171195.kml Cardoso, P.

Supplementary material 6 Distribution of *Scaphidysderinanapo* Platnick & Dupérré, 2011 Data type: Distribution
File: oo_171196.kml Cardoso, P.

Supplementary material 7 Distribution of *Scaphiellatena* Platnick & Dupérré, 2010 Data type: Distribution
File: oo_171197.kml Cardoso, P.

Supplementary material 8 Distribution of *Tapinesthisinermis* (Simon, 1882) Data type: Distribution
File: oo_171198.kml Cardoso, P.

Supplementary material 9 Distribution of *Xyccarphmyops* Brignoli, 1978 Data type: Distribution
File: oo_171199.kml Cardoso, P.

Supplementary material 10 Distribution of *Maoriatamagna* (Forster, 1956) Data type: Distribution
File: oo_171200.kml Cardoso, P.

Supplementary material 11 Distribution of *Tasmanoonopshickmani* Forster & Platnick, 1985 Data type: Distribution
File: oo_171201.kml Cardoso, P.

Supplementary material 12 Distribution of *Hamataliwacordata* Zhang, Zhu & Song, 2005 Data type: Distribution
File: oo_171202.kml Cardoso, P.

Supplementary material 13 Distribution of *Boagriuspumilus* Simon, 1893 Data type: Distribution
File: oo_205355.kml Cardoso, P.

Supplementary material 14 Distribution of *Philodromusgertschi* Schick, 1965 Data type: Distribution
File: oo_171204.kml Cardoso, P.

Supplementary material 15 Distribution of *Suemustibelliformis* Simon, 1909 Data type: Distribution
File: oo_171205.kml Cardoso, P.

Supplementary material 16 Distribution of *Belisanaanhuiensis* (Xu & Wang, 1984) Data type: Distribution
Brief description: Only the southern record representing the type locality is considered relevant.
File: oo_171206.kml Cardoso, P.

Supplementary material 17 Distribution of *Carapoiafowleri* Huber, 2000 Data type: Distribution
File: oo_171207.kml Cardoso, P.

Supplementary material 18 Distribution of *Modisimuscuadro* Huber & Fischer, 2010 Data type: Distribution
File: oo_171166.kml Cardoso, P.

Supplementary material 19 Distribution of *Pholcusjixianensis* Zhu & Yu, 1983 Data type: Distribution
File: oo_203812.kml Cardoso, P.

Supplementary material 20 Distribution of *Smeringopusnatalensis* Lawrence, 1947 Data type: Distribution
File: oo_171209.kml Cardoso, P.

Supplementary material 21 Distribution of *Spermophorajocquei* Huber, 2003 Data type: Distribution
File: oo_171210.kml Cardoso, P.

Supplementary material 22 Distribution of *Charminuscamerunensis* Thorell, 1899 Data type: Distribution
File: oo_171211.kml Cardoso, P.

Supplementary material 23 Distribution of *Dolomedesmendigoetmopasi* Barrion, 1995 Data type: Distribution
File: oo_203813.kml Cardoso, P.

Supplementary material 24 Distribution of *Dolomedestenebrosus* Hentz, 1844 Data type: Distribution
File: oo_205338.kml Cardoso, P.

Supplementary material 25 Distribution of *Hygropodalongitarsis* (Thorell, 1877) Data type: Distribution
File: oo_171215.kml Cardoso, P.

Supplementary material 26 Distribution of *Prodidomuswoodleigh* Platnick & Baehr, 2006 Data type: Distribution
File: oo_171224.kml Cardoso, P.

Supplementary material 27 Distribution of *Theumaaprica* Simon, 1893 Data type: Distribution
File: oo_171218.kml Cardoso, P.

Supplementary material 28 Distribution of *Afraflacilladatuntata* (Logunov & Zamanpoore, 2005) Data type: Distribution
File: oo_203831.kml Cardoso, P.

Supplementary material 29 Distribution of *Attuluszaisanicus* (Logunov, 1998) Data type: Distribution
File: oo_203832.kml Cardoso, P.

Supplementary material 30 Distribution of *Bathippuslatericius* (Thorell, 1881) Data type: Distribution
File: oo_171219.kml Cardoso, P.

Supplementary material 31 Distribution of *Bathippusrechingeri* Kulczyn'ski, 1910 Data type: Distribution
File: oo_171220.kml Cardoso, P.

Supplementary material 32 Distribution of *Brettuscelebensis* (Merian, 1911) Data type: Distribution
File: oo_171221.kml Cardoso, P.

Supplementary material 33 Distribution of *Carrhotusviduus* (C. L. Koch, 1846) Data type: Distribution
File: oo_203839.kml Cardoso, P.

Supplementary material 34 Distribution of *Corythaliatropica* (Mello-Leitão, 1939) Data type: Distribution
File: oo_205349.kml Cardoso, P.

Supplementary material 35 Distribution of *Grayenullaaustralensis* Zabka, 1992 Data type: Distribution
File: oo_171415.kml Cardoso, P.

Supplementary material 36 Distribution of *Heliophanuscapicola* Simon, 1901 Data type: Distribution
File: oo_171416.kml Cardoso, P.

Supplementary material 37 Distribution of *Helvetiastridulans* Ruiz & Brescovit, 2008 Data type: Distribution
File: oo_171417.kml Cardoso, P.

Supplementary material 38 Distribution of *Jerzegobipartitus* (Simon, 1903) Data type: Distribution
File: oo_203847.kml Cardoso, P.

Supplementary material 39 Distribution of *Menemerusmirabilis* Wesolowska, 1999 Data type: Distribution
File: oo_171427.kml Cardoso, P.

Supplementary material 40 Distribution of *Myrmarachnebicolor* (L. Koch, 1879) Data type: Distribution
File: oo_205339.kml Cardoso, P.

Supplementary material 41 Distribution of *Myrmarachnepaviei* (Simon, 1886) Data type: Distribution
File: oo_171428.kml Cardoso, P.

Supplementary material 42 Distirbution of *Noeguspetrusewiczi* Caporiacco, 1947 Data type: Distribution
File: oo_171431.kml Cardoso, P.

Supplementary material 43 Distribution of *Parahelpisabnormis* (Zabka, 2002) Data type: Distribution
File: oo_171432.kml Cardoso, P.

Supplementary material 44 Distribution of *Pelegrinaaeneola* (Curtis, 1892) Data type: Distribution
File: oo_171433.kml Cardoso, P.

Supplementary material 45 Distribution of *Pharacocerusfagei* Berland & Millot, 1941 Data type: Distribution
File: oo_203548.kml Cardoso, P.

Supplementary material 46 Distribution of *Phidippusworkmani* Peckham & Peckham, 1901 Data type: Distribution
File: oo_205348.kml Cardoso, P.

Supplementary material 47 Distribution of *Plexippusclemens* (O. Pickard-Cambridge, 1872) Data type: Distribution
File: oo_203558.kml Cardoso, P.

Supplementary material 48 Distribution of *Pristobaeusclarus* (Roewer, 1938) Data type: Distribution
File: oo_203853.kml Cardoso, P.

Supplementary material 49 Distribution of *Rhenecancer* Wesolowska & Cumming, 2008 Data type: Distribution
File: oo_171446.kml Cardoso, P.

Supplementary material 50 Distribution of *Rhenehinlalakea* Barrion & Litsinger, 1995 Data type: Distribution
File: oo_171447.kml Cardoso, P.

Supplementary material 51 Distribution of *Rheneipis* Fox, 1937 Data type: Distribution
File: oo_205347.kml Cardoso, P.

Supplementary material 52 Distribution of *Salticustricinctus* C. L. Koch, 1846 Data type: Distribution
File: oo_203562.kml Cardoso, P.

Supplementary material 53 Distribution of *Sidusacambridgei* (Chickering, 1946) Data type: Distribution
File: oo_203593.kml Cardoso, P.

Supplementary material 54 Distribution of *Synagelidesgosainkundicus* Bohdanowicz, 1987 Data type: Distribution
File: oo_171452.kml Cardoso, P.

Supplementary material 55 Distribution of *Synemosynaaurantiaca* (Mello-Leitão, 1917) Data type: Distribution
File: oo_171453.kml Cardoso, P.

Supplementary material 56 Distribution of *Taualaminutus* Wanless, 1988 Data type: Distribution
File: oo_171455.kml Cardoso, P.

Supplementary material 57 Distribution of *Thyeneaustralis* Peckham & Peckham, 1903 Data type: Distribution
File: oo_171461.kml Cardoso, P.

Supplementary material 58 Distribution of *Titanattuspaganus* Chickering, 1946 Data type: Distribution
File: oo_171462.kml Cardoso, P.
